# Association between genetically determined telomere length and health‐related outcomes: A systematic review and meta‐analysis of Mendelian randomization studies

**DOI:** 10.1111/acel.13874

**Published:** 2023-05-26

**Authors:** Boran Chen, Yushun Yan, Haoran Wang, Jianguo Xu

**Affiliations:** ^1^ Department of Neurosurgery West China Hospital of Sichuan University Chengdu China; ^2^ Psychiatric Laboratory and Mental Health Center, The State Key Laboratory of Biotherapy West China Hospital of Sichuan University Chengdu China; ^3^ Department of Burns and Plastic Surgery West China Hospital of Sichuan University Chengdu China

**Keywords:** health‐related outcomes, leukocyte telomere length, Mendelian randomization, meta‐analysis, systematic review

## Abstract

Emerging evidence has shown that leukocyte telomere length (LTL) is associated with various health‐related outcomes, while the causality of these associations remains unclear. We performed a systematic review and meta‐analysis of current evidence from Mendelian randomization (MR) studies on the association between LTL and health‐related outcomes. We searched PubMed, Embase, and Web of Science up to April 2022 to identify eligible MR studies. We graded the evidence level of each MR association based on the results of the main analysis and four sensitive MR methods, MR‐Egger, weighted median, MR‐PRESSO, and multivariate MR. Meta‐analyses of published MR studies were also performed. A total of 62 studies with 310 outcomes and 396 MR associations were included. Robust evidence level was observed for the association between longer LTL and increased risk of 24 neoplasms (the strongest magnitude for osteosarcoma, GBM, glioma, thyroid cancer, and non‐GBM glioma), six genitourinary and digestive system outcomes of excessive or abnormal growth, hypertension, metabolic syndrome, multiple sclerosis, and clonal hematopoiesis of indeterminate potential. Robust inverse association was observed for coronary heart disease, chronic kidney disease, rheumatoid arthritis, juvenile idiopathic arthritis, idiopathic pulmonary fibrosis, and facial aging. Meta‐analyses of MR studies suggested that genetically determined LTL was associated with 12 neoplasms and 9 nonneoplasm outcomes. Evidence from published MR studies supports that LTL plays a causal role in various neoplastic and nonneoplastic diseases. Further research is required to elucidate the underlying mechanisms and to bring insight into the potential prediction, prevention, and therapeutic applications of telomere length.

## Graphical abstract

This systematic review and meta‐analysis found that genetically determined telomere length was robustly associated with increased risk of neoplasms, hypertension, metabolic syndrome, multiple sclerosis, and clonal hematopoiesis of indeterminate potential and decreased risk of coronary heart disease, chronic kidney disease, rheumatoid arthritis, juvenile idiopathic arthritis, idiopathic pulmonary fibrosis, and facial aging.

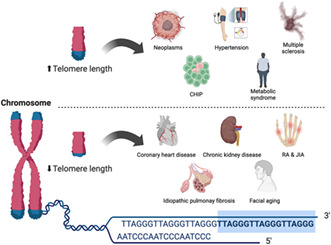

## 1 BACKGROUND

Telomeres are protein‐DNA complexes at the ends of linear eukaryotic chromosomes that maintain genomic stability and integrity (Chan & Blackburn, 2004). Telomeres consist of repeated “TTAGGG” sequences and shorten steadily with each cell replication (O'Sullivan & Karlseder, 2010). When telomeres are critically short, replicative senescence, genomic instability, and apoptosis arise (Blasco, 2005; Gasser, 2000; O'Sullivan &Karlseder, 2010). Leukocyte telomere length (LTL), which is the proxy for telomere length across different tissues, has been studied extensively and proposed as a biomarker of human aging (Demanelis et al., 2020; López‐Otín et al., 2013). Observational studies have shown that LTL is associated with the risk of various age‐related diseases such as cardiovascular diseases and Type 2 diabetes mellitus, some types of cancer, and the human life span (Arbeev et al., 2020; Haycock et al., 2014; Rode et al., 2016; Wentzensen et al., 2011; Willeit et al., 2014). However, the nature of observational studies makes the observed association between LTL and outcomes susceptible to reverse causation and unmeasured confounding (Sekula et al., 2016).

Mendelian randomization (MR) is an instrumental variable method using single‐nucleotide polymorphisms (SNPs) as the instrumental variable to make causal inferences between modifiable exposures and outcomes in nonexperimental data (Burgess et al., 2017; Davey Smith & Hemani, 2014). The alleles of a given SNP are randomly allocated to gametes through meiosis and are therefore not generally vulnerable to reverse causation and residual confounding, which mimics the randomized allocation of the exposure in the population (Sekula et al., 2016). A genetic variant must satisfy the following assumptions to be used for the causal effect estimation: (i) the SNP is associated with the exposure (the relevance assumption), (ii) the SNP is independent of common causes of the outcome (the independent assumption), (iii) the SNP affects the outcome only through the exposure (the exclusion restriction assumption) (Taylor et al., 2014). When the genetic variant is valid, causal effect can be unbiasedly assessed.

The published genome‐wide association studies (GWASes) have provided large‐scale genetic information and advanced our understanding of genetic variants associated with LTL (Codd et al., 2013; Codd et al., 2021; Dorajoo et al., 2019; Li et al., 2020; Mangino et al., 2012; Pooley et al., 2013). Furthermore, the number of MR studies utilizing genetic data from GWASes has substantially increased during the past decade, which have yielded inconsistent findings. This systematic review and meta‐analysis aimed to map the causal associations between LTL and various health‐related outcomes and pool the results of published MR studies.

## 2 METHODS

This study was reported following the Preferred Reporting Items for Systematic Review and Meta‐Analysis (PRISMA) checklist.

### 2.1 Search strategy and selection criteria

We performed a comprehensive literature search in the PubMed, Embase, and Web of Science databases from inception through April 13, 2022 to identify all MR studies exploring the association between genetically determined LTL and any health‐related outcome. The following search items were used: “telomere” OR “telomere length” OR “leukocyte telomere length” OR “LTL” for the exposure and “Mendelian randomization” OR “instrumental variable analysis” for the study design. Detailed search strategy is shown in Supplementary Method. We also identified potentially eligible studies according to the reference lists of included studies and relevant reviews.

Studies to be included needed to (1) use MR; (2) use LTL as the exposure; and (3) measure the causal association between the exposure and one or more health‐related outcomes. Studies met any of the following criteria were excluded: (1) nonhuman studies; (2) conference abstracts, editorials, or reviews; (3) not written in English or Chinese; or (4) full text was not available. If more than one study reported the same outcome in the same population, we included the one with the most participants.

All articles were exported to Endnote (version 20.2, Clarivate Analytics) after the literature search and duplicated records were removed. Two independent reviewers (BC and YY) assessed the articles according to the inclusion and exclusion criteria, and any disagreement was arbitrated by a third reviewer (HW). Articles were first selected by titles and abstracts and then full texts.

### 2.2 Data extraction and quality assessment

One author (BC) extracted the information from each article and assessed the quality of included studies, and the extracted information and quality assessment were checked by two authors (YY and HW). The following information was extracted and filled into the predefined tables: the family name of the first author, year of publication, study design (individual‐level or summary‐level MR), the number of instrumental variables, sample size (exposure and outcome), exposure (longer or shorter telomere length), health‐related outcome, analysis for effect estimation, odds ratio (OR) or hazard ratio (HR) per 1 standard deviation (SD) increase in telomere length with 95% CI and *p* value (for studies reported OR per 1 kb increase or others, the effect size was rescaled to 1 SD increase in telomere length) for the main and sensitive analyses, data source, ancestry, and quality‐associated information (tests for the relevance, independence, and exclusion restriction assumptions).

There are no formal tools for the assessment of the risk of bias for MR studies. The quality of studies included in the current systematic review was discussed according to the main analysis to estimate the effect size of telomere length on health‐related outcomes as well as the tests of three key assumptions of each MR study.

### 2.3 Evaluation of evidence level

We evaluated the evidence level for causality through the criteria raised by Markozannes et al. (2022). Briefly, MR associations were graded based on the main analysis and four commonly used sensitive analyses, MR Egger, weighted median (WM), MR pleiotropy residual sum, and outlier test (MR‐PRESSO), and multivariable MR (MVMR). The MR Egger method can detect directional horizontal pleiotropy and make causal inferences that are not subject to the pleiotropy, although it compromises power (Bowden et al., 2015). The WM method provides consistent estimates even when up to 50% of the weight was contributed by invalid instrumental variables (Bowden et al., 2016). MR‐PRESSO can detect outliers and make effect estimations after removing outliers (Verbanck et al., 2018). MVMR is an extension of univariate MR and allows joint detection of causal effects of multiple risk factors (Burgess & Thompson, 2015). In the grading system, if adjustment for multiple tests was reported for the main analysis, the adjusted *p* value threshold was used. For the sensitive analysis, *p* value of 0.05 was considered significant. An MR association was graded non‐evaluable when only the main analysis was performed. For evaluable associations, when all the methods achieved statistical significance and the direction of the effect sizes were concordant, the evidence level was graded robust. When at least one method achieved statistical significance and the direction of the effect sizes were concordant, the evidence level was graded probable. When at least one method achieved statistical significance and the direction of the effect sizes were not concordant, the evidence level was graded suggestive. When none of the methods achieved statistical significance, the evidence level was graded insufficient. We also performed an additional grading by excluding the MR Egger method from the analysis due to the low power.

### 2.4 Statistical analysis

Stata (version 16.0, Stata Corporation) software was used to perform meta‐analysis of included MR associations. ORs per 1 SD increase in telomere length with 95% CIs for each health‐related outcome were pooled using a random‐effect model. Only the outcomes reported by two or more studies and with the same ancestry were analyzed. Associations with overlapping population were not pooled. Because there were no restrictions on validation for the key assumptions of MR, we performed sensitivity analyses by removing studies that did not select instrumental variables from GWAS, studies without tests for horizontal pleiotropy, or studies with evidence of pleiotropy. The heterogeneity between studies was determined by *I* ^2^ test. *I* ^2^ < 25%, between 25% and 50%, between 50% and 75%, and >75% was considered low, mild, moderate, and high heterogeneity, respectively. *p*‐Values were corrected for multiple testing via false discovery rate (FDR). FDR‐corrected *p* < 0.05 was considered statistically significant.

## 3 RESULTS

### 3.1 Literature search

**FIGURE 1:**
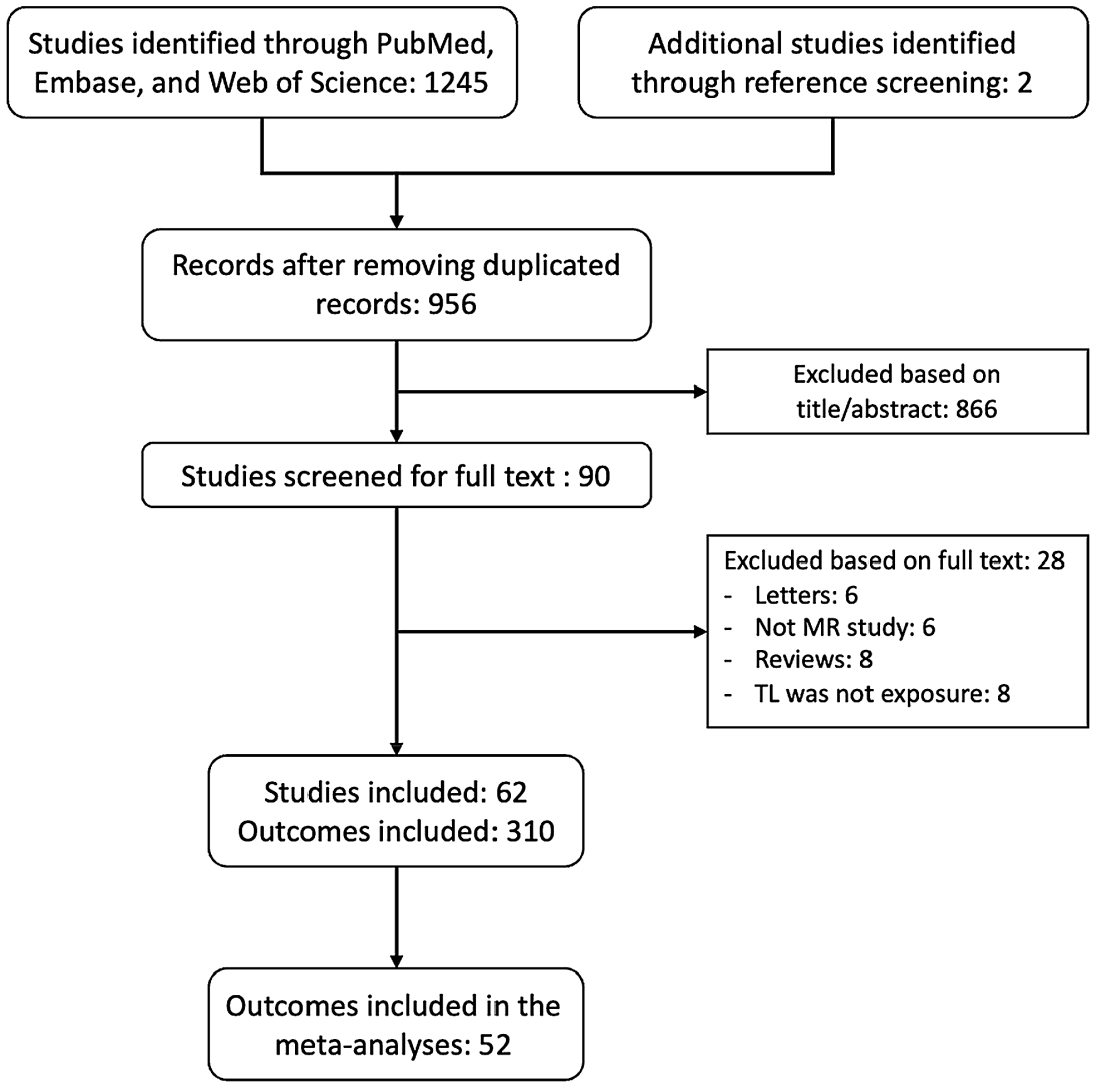
Flow chart of the systematic review and meta‐analysis.

The initial search yielded 1245 records, of which 954 remained after removing duplicates. Sixty‐two MR studies with 310 outcomes were enrolled in the systematic review and 52 outcomes were included in the meta‐analysis (Figure 1).

### 3.2 Study characteristics and quality description

Most of the studies (51/62, 82.3%) used summary‐level MR approach, 26 (41.9%) studies used individual‐level approach, and 15 (24.2%) studies used both individual‐level and summary‐level MR approaches. The first MR study was published in 2012, while the majority were published after 2019 (39/62, 62.9%). Most studies (49/62, 79.0%) included participants of European ancestry, while the remaining studies included East Asian (or Asian), Black, Hispanic, or mixed individuals. One study did not state the ancestry of participants.

All studies used full instrumental variable analyses, such as the use of weighted genetic score for individual‐level MR studies and inverse variance weighted for summary‐level MR studies. Three of the included studies were GWASes of LTL with MR analyses exploring the causal links between LTL and disease outcomes. The remaining studies all identified instrumental variables from published GWASes of LTL, except for one that identified SNPs based on their biological function. The significance level for identifying instrumental variables was 5 × 10^−8^ in most studies, while several studies did not report the significance level. F‐statistics were reported in 19 studies (30.6%), which were all above 10 except 1, indicating the presence of weak instrument bias. All the individual‐level MR studies adjusted for potential confounders, while 29 (56.9%) of the summary‐level MR studies did not report the adjustment. However, this did not mean the violation of the second assumption, because covariates might have been adjusted in the GWASes of outcomes and genetic instruments are not susceptible to potential confounders due to the random allocation at conception. There is no appropriate strategy to test pleiotropy in individual‐level MR analyses. As a result, pleiotropy was tested in only a few individual‐level studies by excluding potentially pleiotropic SNPs. In contrast to individual‐level MR studies, most summary‐level studies (43/48, 84.3%) tested the pleiotropy by “gtx” package, heterogeneity, asymmetry of funnel plots, goodness‐of‐fit test, or more formal techniques such as MR Egger and MR‐PRESSO. Detailed description of the study quality is shown in Table S1.

### 3.3 Evidence level and meta‐analyses results

A total of 396 associations were included in the systematic review, with 258 (65.2%) of them not graded because only the main analysis was performed. Among these non‐evaluable associations, 24 (9.3%) used an individual‐level approach, 172 (66.7%) used a summary‐level approach, and 62 (24.0%) used both individual‐level and summary‐level approaches. Thirty‐five (13.6%) of the non‐evaluable associations had a statistically significant main analysis result. In the graded 138 (34.8%) associations, there were 40 (30.0%, 10.1% of total) with robust evidence, 34 (24.6%, 8.6% of total) with probable evidence, 2 (1.4%, 0.5% of total) with suggestive evidence, and 62 (44.9%, 15.7% of total) with insufficient evidence (Table 1). WM was the most used sensitive analysis method, of which 50.9% (57/112) were statistically significant. MR Egger and MR‐PRESSO were performed in 64 and 16 analyses, of which 17 (26.6%) and 8 (50.0%) were statistically significant, respectively. Only one analysis performed MVMR, which presented a statistically significant result (Table S2). After removing results of the MR Egger method from the grading criteria, nine associations with probable evidence and one association with suggestive evidence were upgraded to robust. Two associations with probable evidence were downgraded to insufficient. In addition, 21 associations with MR Egger as the sole sensitive analysis were not graded (Tables S3 and S4).

**TABLE 1:** Number and percentage of MR associations per evidence level by outcome category.

Outcome category	Robust evidence	Probable evidence	Suggestive evidence	Insufficient evidence	Non‐evaluable	Total
Cardiovascular	2 (8.3)	3 (12.5)	0 (0)	1 (4.2)	18 (75)	24 (100)
Digestive	1 (4.6)	2 (9.1)	1 (4.6)	0 (0)	18 (81.8)	22 (100)
Endocrine	1 (5.6)	4 (22.2)	0 (0)	1 (5.6)	12 (66.7)	18 (100)
Genitourinary	6 (46.2)	1 (7.7)	0 (0)	2 (15.4)	4 (30.8)	13 (100)
Immune	1 (4.6)	1 (4.6)	0 (0)	1 (4.6)	19 (86.4)	22 (100)
Infections	0 (0)	0 (0)	0 (0)	1 (6.7)	14 (93.3)	15 (100)
Mortality	0 (0)	0 (0)	0 (0)	0 (0)	46 (100)	46 (100)
Musculoskeletal	1 (4.8)	1 (4.8)	1 (4.8)	9 (42.9)	9 (42.9)	21 (100)
Neoplasm	24 (16.9)	17 (12)	0 (0)	31 (21.8)	70 (49.3)	142 (100)
Neurological/psychiatric	1 (2.6)	3 (7.7)	0 (0)	7 (18)	28 (71.8)	39 (100)
Respiratory	1 (4.8)	1 (4.8)	0 (0)	6 (28.6)	13 (61.9)	21 (100)
Sensory	0 (0)	1 (12.5)	0 (0)	1 (12.5)	6 (75)	8 (100)
Other	2 (40)	0 (0)	0 (0)	2 (40)	1 (20)	5 (100)
Total	40 (10.1)	34 (8.6)	2 (0.5)	62 (15.7)	258 (65.2)	396 (100)

**FIGURE 2:**
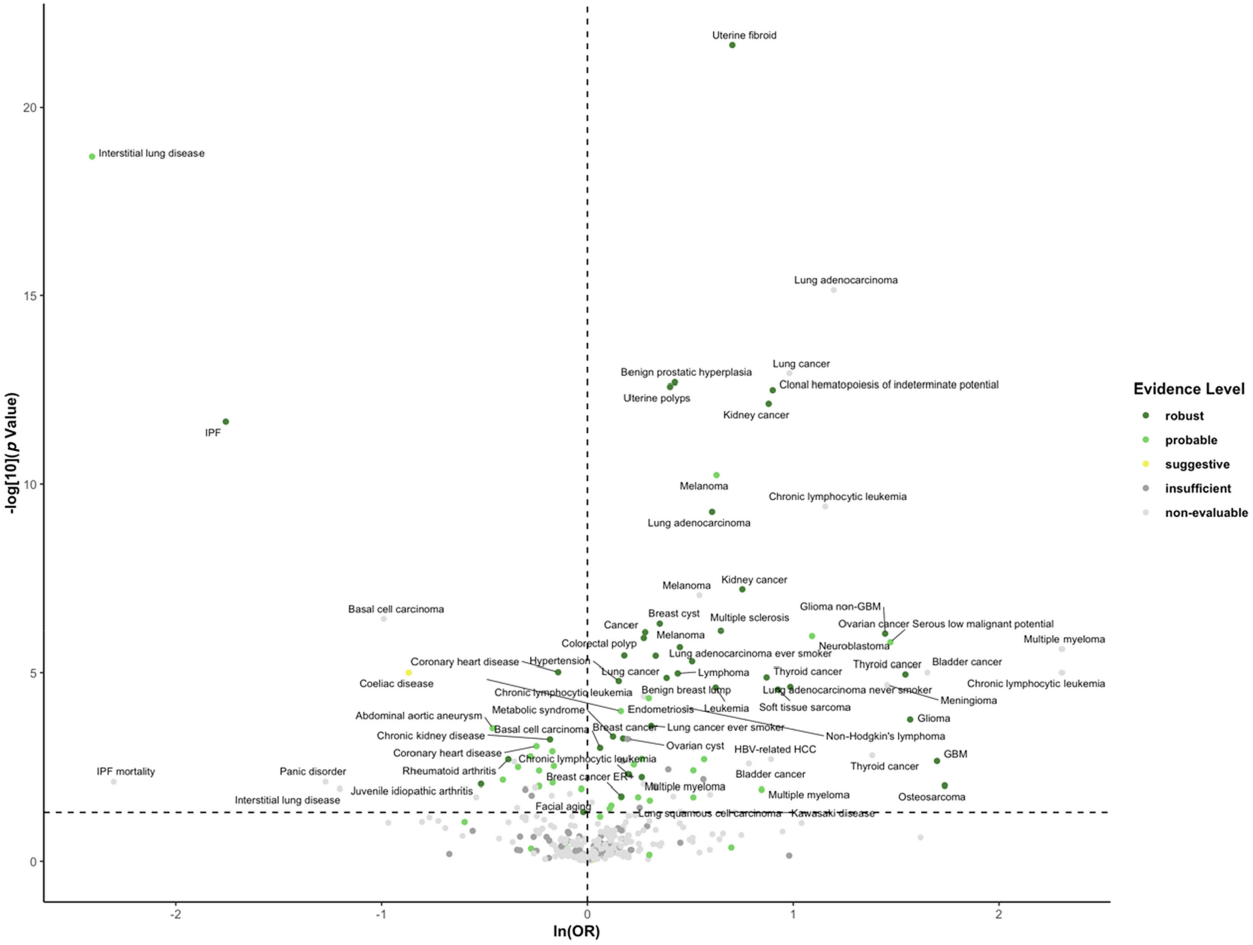
Volcano plot of odds ratio (OR) and *p*‐value of the association between genetically determined longer leukocyte telomere length (LTL) and the risk of health‐related outcomes. ORs and *p*‐value of the main analysis are used. ORs are rescaled to 1 SD increase in LTL. Associations with an OR that cannot be rescaled are not presented. The horizontal and vertical dashed lines correspond to *p* = 0.05 and OR = 1, respectively.

**FIGURE 3:**
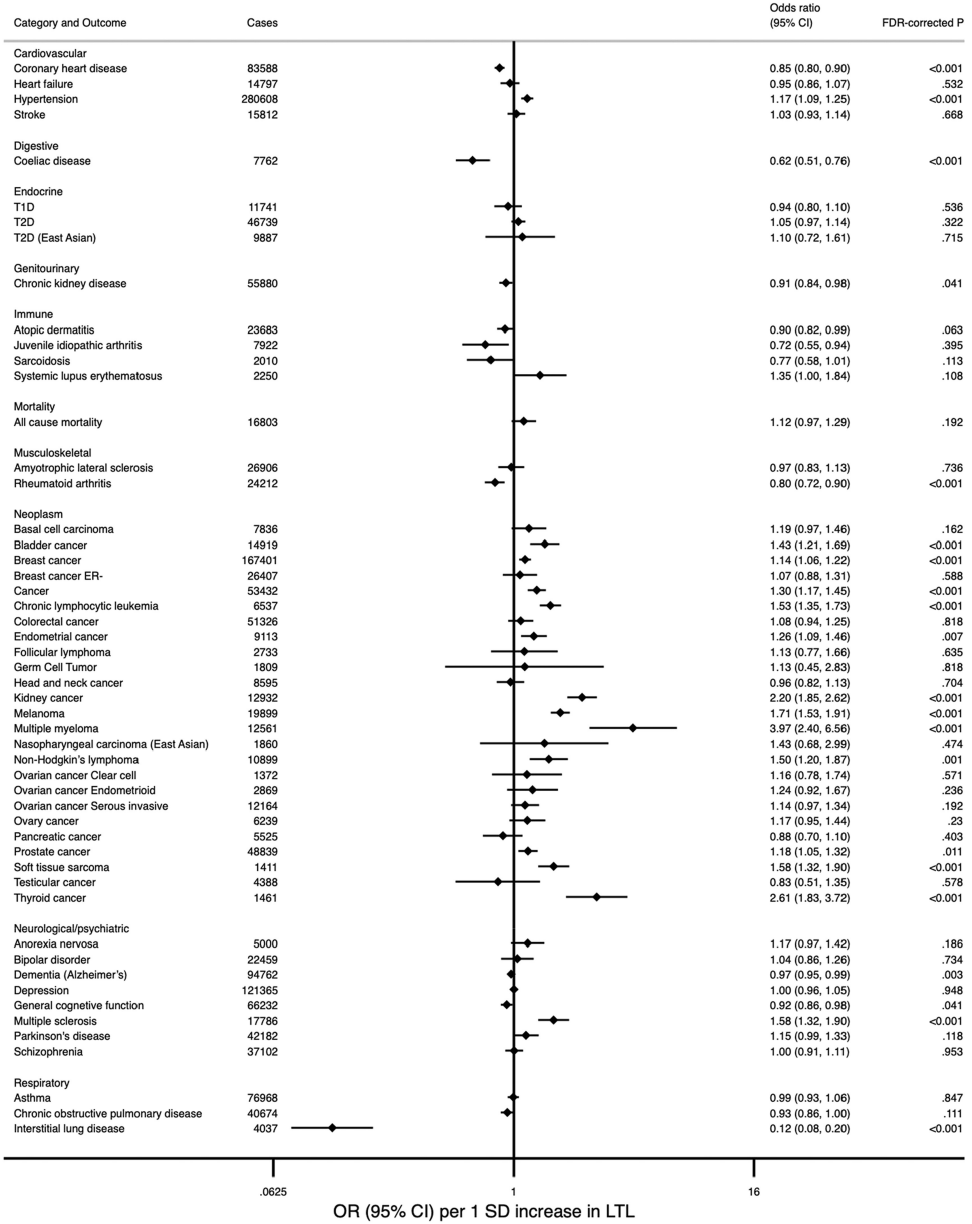
Meta‐analyses results for the association of genetically longer leukocyte telomere length (LTL) with health‐related outcomes. Odds ratios and the corresponding 95% CIs per 1 SD increase in LTL are presented. *p*‐Values are FDR corrected, and an FDR corrected *p* < 0.05 is considered statistically significant.

The strength of the association between genetically determined LTL and health‐related outcomes as well as the evidence level of each association are shown in Figure 2, with corresponding tabulated data in Table S3. Neoplasms had the highest number of robust associations, pertaining to increased risk of osteosarcoma, glioma, glioblastoma (GBM), non‐GBM glioma, thyroid cancer (*N* = 2), lung cancer (*N* = 2), lung adenocarcinoma (*N* = 2), soft tissue sarcoma, kidney cancer (*N* = 2), leukemia, chronic lymphocytic leukemia, melanoma, lymphoma, multiple myeloma, all cancers (*N* = 2), breast cancer, ER‐positive breast cancer, and basal cell carcinoma. Additionally, the associations for adolescent‐onset ependymoma, neuroblastoma, and serous low‐malignant‐potential ovarian cancer were upgraded to robust after removing MR Egger (Figure 2 and Table S3). In the meta‐analyses, genetically longer LTL was associated with increased risk of multiple myeloma (3.97 [2.40–6.56]), thyroid cancer (2.61 [1.83–3.72]), kidney cancer (2.20 [1.86–2.62]), melanoma (1.71 [1.53–1.91]), soft tissue sarcoma (1.59 [1.33–1.90]), chronic lymphocytic leukemia (1.53 [1.35–1.73]), all cancers (1.30 [1.17–1.45]), breast cancer (1.14 [1.06–1.22]), non‐Hodgkin's lymphoma (1.50 [1.20–1.87]), bladder cancer (1.43 [1.21–1.69]), endometrial cancer (1.26 [1.09–1.46]), and prostate cancer (1.18 [1.05–1.32]). However, LTL was not significantly associated with the risk of basal cell carcinoma (1.19 [0.97–1.46]) (Figure 3 and Table S5). We observed substantial viability of association for neoplasms within the same tissue site. For example, LTL was robustly correlated with ER‐positive breast cancer, while the two associations for ER‐negative breast cancer were graded insufficient and non‐evaluable (with non‐significant main analysis result). In addition, the evidence level for lung adenocarcinoma was robust, while that for lung squamous cell carcinoma or small cell lung carcinoma was insufficient or non‐evaluable. After removing studies that did not select instrumental variables from GWAS, studies without tests for horizontal pleiotropy, or studies with evidence of pleiotropy, the association for basal cell carcinoma turned significant (1.30 [1.05–1.61]), while there was minor impact on other associations (Table S6).

Among the 13 analyses for genitourinary system outcomes, 6 (46.2%) were robust, pertaining to increased risk of benign breast lump, benign prostatic hyperplasia, breast cyst, uterine fibroid, uterine polyps, and decreased risk of chronic kidney disease (Figure 2 and Table S3). Meta‐analysis showed an association between genetically longer LTL and decreased risk of chronic kidney disease (0.91 [0.85–0.98]) with high heterogeneity (*I* ^2^ = 83.9%), while analyses for other outcomes were not pooled (Figure 3 and Table S5).

We found two robust associations in cardiovascular system outcomes, pertaining to longer LTL with increased risk of hypertension and decreased risk of coronary heart disease (Figure 2 and Table S3). In the meta‐analyses, genetically determined longer LTL was also significantly associated with increased risk of hypertension (1.17 [1.09–1.25]) and decreased risk of coronary heart disease (0.85 [0.80–0.90]) (Figure 3 and Table S5). In the sensitive meta‐analyses, there was minor impact on the association for coronary heart disease (0.85 [0.81–0.90]) (Table S6).

We observed one robust association, longer LTL with decreased risk of idiopathic pulmonary fibrosis (IPF), in respiratory system outcomes. Additionally, longer LTL with decreased risk of interstitial lung disease (ILD) was upgraded from probable to robust evidence after removing MR Egger (Figure 2 and Table S3). In the meta‐analyses, genetically determined longer LTL was associated with decreased risk of ILD (0.12 [0.08–0.20]) (Figure 3 and Table S5).

Only one robust association in endocrine system pertained to metabolic syndrome. Additionally, longer LTL with increased risk of chronic kidney disease in T2D patients was upgraded from probable to robust evidence after removing MR Egger (Figure 2 and Table S3). Meta‐analysis did not find significant association between genetically determined LTL and T1D or T2D (Figure 3 and Table S5).

For neurological/psychiatric outcomes, the association for multiple sclerosis was robust. After excluding MR Egger, dementia (Alzheimer's) was upgraded to robust (Figure 2 and Table S3). Meta‐analyses indicated that genetically longer LTL was associated with decreased risk of dementia (Alzheimer's) (0.97 [0.95–0.99]), general cognitive function (0.92 [0.86–0.99]), and increased risk of multiple sclerosis (1.58 [1.33–1.90]) (Figure 3 and Table S5). Sensitive meta‐analysis showed that the association for multiple sclerosis remained (Table S6).

Two robust associations for digestive system outcomes pertained to increased risk of colorectal polyp and decreased risk of celiac disease (after excluding MR Egger) (Figure 2 and Table S3). In the meta‐analyses, genetically determined longer LTL was associated with decreased risk of celiac disease (0.62 [0.51–0.76]), with high heterogeneity between studies (*I* ^2^ = 81.2%) (Figure 3 and Table S5).

For outcomes of other systems, the associations for rheumatoid arthritis and juvenile idiopathic arthritis were robust, with corresponding meta‐analyses results of 0.80 (0.72–0.90) and 0.72 (0.55–0.94), respectively. Other robust associations were those for clonal hematopoiesis of indeterminate potential and facial aging (Figures 2 and 3 and Tables S3 and S5). There were no robust associations between LTL and sensory outcomes, infections, and mortalities (Table 1).

## 4 DISCUSSION

This systematic review and meta‐analysis included a total of 62 studies with 310 outcomes and 396 MR associations investigating the causal relationships between LTL and various health‐related outcomes. We categorized the evidence level of each MR association based on the results of the main analysis as well as four sensitive analyses, MR Egger, WM, MR‐PRESSO, and MVMR. We observed 40 robust MR associations, with the highest number in neoplasms and highest percentage in genitourinary outcomes. Each category in cardiovascular, respiratory, endocrine, neurological/psychiatric, digestive, musculoskeletal, and immune system had one or two robust MR associations. The meta‐analyses suggested that genetically determined LTL was associated with 12 neoplasms and 9 non‐neoplasm outcomes.

We found that genetically predicted LTL was consistently associated with increased risk of neoplasms, which contrasted with previous observational reports (Zhang et al., 2017; Zhu et al., 2016). We also found increased risk of genitourinary system outcomes of excessive or abnormal growth. The underlying mechanism might be that cells with longer telomeres or the ability of telomere maintenance, such as the telomerase and the alternative lengthening of telomeres (ALT) pathway, have increased replicative potential and a greater chance to acquire mutations that occur during DNA replication (De Vitis et al., 2018). The magnitude of association varied among neoplasms of different tissues or those within the same tissue site. This could be explained by the discrepancy in the number of stem cell divisions across different tissues and the variation in the biological characteristics of different subtypes within the same tissue site (Gao et al., 2020). Genetic variants near TERT, TERC, or OBFC1 may have independent impact on the risk of certain types of cancer, which violates the exclusion restriction assumption of MR (Zheng et al., 2019). However, several MR studies have found that the association was unlikely to be attributable to pleiotropy (Campa et al., 2019; Kachuri et al., 2019; Machiela et al., 2016; Walsh et al., 2015).

Robust evidence for the inverse association between LTL and chronic kidney disease as well as chronic kidney disease in T2D patients was in accordance with observational studies (Raschenberger et al., 2015; Tentolouris et al., 2007). It was postulated that deregulated renin‐angiotensin‐aldosterone system promotes increased burden of oxidative stress and inflammation, resulting in telomere length shortening and kidney damage (Vasan et al., 2008). Thus, telomere length was believed to be an indicator and biomarker of chronic kidney disease. However, the MR analysis provides robust evidence for the causal role of telomere length on chronic kidney disease, suggesting that telomere length may be a potential therapeutic target in chronic kidney disease.

Two cardiovascular system outcomes were robustly correlated with LTL, including decreased risk of coronary heart disease and increased risk of hypertension. Results of the meta‐analyses also confirmed these associations. Coronary heart disease is a degenerative disease. Shortened telomeres might contribute to the accumulation of senescent endothelial cells and vascular smooth muscle cells, which exacerbate atherosclerosis and inflammation that play key roles in the formation of coronary heart disease (Aschacher et al., 2017). The pooled result of MR studies for hypertension was the opposite of the observational association, possibly due to residual bias in observational studies (Codd et al., 2021). In addition, Kuo et al. (2019) reported that the association was stronger in adults aged over 60 years than in younger adults. However, the mechanism of the association between genetically longer LTL and increased risk of hypertension is unclear and needs further investigation.

The association between genetically determined LTL and two respiratory system outcomes, that is, ILD and IPF, was robust and strong, with genetically 1 SD shorter LTL increasing the risk of both outcomes by over five times. Our finding was in accordance with observational result of the inverse association between telomere length and IPF (Dai et al., 2015). IPF is the major category of ILD, characterized by extracellular matrix accumulation and aberrant remodeling of lung interstitium (Richeldi et al., 2017). Evidence has shown that telomere dysfunction causes cellular senescence of alveolar stem cells and Type 2 alveolar epithelial cells, which provokes inflammation by triggering up‐regulation of immune‐signaling pathways and contributes to the susceptibility to injury by environmental risk factors such as smoking, occupational exposures, viral infection, mechanical strain, and air pollution (Alder et al., 2015). Thus, telomere shortening drives IPF via both intrinsic and extrinsic mechanisms.

Genetically predicted LTL was associated with several outcomes of other systems, including metabolic syndrome, multiple sclerosis, colorectal polyp, coeliac disease, rheumatoid arthritis, juvenile idiopathic arthritis, clonal hematopoiesis of indeterminate potential, and facial aging. Meta‐analyses suggested significant association between longer LTL and decreased risk of dementia (Alzheimer's) (0.97 [0.95–0.99]), which was upgraded to robust after excluding MR Egger, and impaired general cognitive function (0.92 [0.86–0.99]). However, the evidence level of two MR analyses for general cognitive function were non‐evaluable. A previous study investigated the causal relationship of longer LTL on metabolic syndrome and found that the forward association was primarily driven by blood pressure and upper‐body fat distribution (Loh et al., 2021). An in vivo model also linked metabolic syndrome components to telomere length (Muñoz‐Lorente et al., 2019). Results of MR studies for multiple sclerosis were inconsistent with observational studies that reported an inverse association (Bühring et al., 2021). The mechanism by which longer LTL increased the risk of multiple sclerosis needs further investigation. However, the results should be interpreted taking into account possible nonlinear relationships (Shu et al., 2022). Decreased telomere was associated with increased risk of coeliac disease in observational studies, which is in the same direction as the genetic evidence (Kamycheva et al., 2017). The mechanisms behind the association remain unclear, while authors of these observational studies hypothesized that oxidative stress or chronic inflammation stress of these diseases causes telomere shortening (Kamycheva et al., 2017). Whether there is bidirectional causality between telomere length and these outcomes needs to be further investigated. The meta‐analysis result of general cognitive function was in line with observational findings (Hägg et al., 2017; Ma et al., 2013; Valdes et al., 2010; Yaffe et al., 2011). Telomere shortening may lead to changes of functional connectivity and structural connectivity that modulate cognition, albeit with plausible bidirectional causality (Yu et al., 2020). Results of MR studies for rheumatoid arthritis and juvenile idiopathic arthritis were in accordance with observational evidence (Picarelli et al., 2017; Zeng et al., 2020; Zhang, 2022). Studies have shown that loss of telomere might lead to immune‐deficiency, loss of peripheral tolerance, adaptive immunity dy dysfunction, and infection. Furthermore, shortened telomeres might contribute to excessive inflammatory cytokine production, leading to joint destruction and systemic symptoms (Zeng et al., 2020; Zhang, 2022).

Evidence of the causal role of LTL in these outcomes, whether increased or decreased, suggested potential prediction, prevention, and treatment applications of telomere length. By measuring LTL, information on the risk of developing neoplastic or nonneoplastic diseases for individuals can be obtained. In addition, telomerase activation therapy may help with some nonneoplastic diseases that were caused by shortened telomeres, but it can also lead to carcinogenesis (Ramunas et al., 2015). Healthy lifestyles such as exercise, reduction of life stress, and mindfulness can promote telomere length, which could be encouraged in individuals with shortened telomere (Duckworth et al., 2021). However, the trade‐off between neoplastic and nonneoplastic diseases must be considered.

This study has some limitations. First, the grading criteria of evidence level is more inclined to evaluate associations using summary‐level approaches, 24 associations without MR Egger, WM, MR‐PRESSO, and MVMR were not graded. This also highlights the need for a more thorough assessment of sensitive MR analyses for future MR studies. Second, we investigated the causal effect of LTL on health‐related outcomes, not telomere length detected in disease‐relevant tissues. However, telomere length within an individual is less variable than among individuals and LTL can serve as a proxy for telomere length across various tissues (Demanelis et al., 2020; López‐Otín et al., 2013). Third, outcomes that were reported by only one study were not included in the meta‐analysis. Moreover, the results of several studies were not pooled because they used categorical metrics for LTL, or OR was reported per 10% decrease in LTL. Fourth, we assumed a linear association and therefore were not able to find if there was “J” or “U” shaped association. Fifth, this is a study‐level meta‐analysis, with high heterogeneity among studies for chronic lymphocytic leukemia, chronic kidney disease, bladder cancer, soft tissue sarcoma, and celiac disease. Finally, most studies were comprised of individuals of European ancestry, thus the results may not generalize to non‐European populations.

In conclusion, evidence from published MR studies supports that LTL plays a causal role in various neoplastic and nonneoplastic diseases. Further research is required to elucidate the underlying mechanisms and to bring insight into the potential prediction, prevention, and therapeutic applications of telomere length.

## AUTHOR CONTRIBUTIONS

Boran Chen and Jianguo Xu conceived and supervised the study. Boran Chen, Yushun Yan, and Haoran Wang contributed to literature searching, study screening, and data collection. Boran Chen and Yushun Yan performed data analysis. Boran Chen, Yushun Yan, and Jianguo Xu wrote and edited the manuscript. All authors approved the final version of the manuscript.

## FUNDING INFORMATION

This study was funded by General Program of the National Natural Science Foundation of China (82173175) and Knowledge Innovation Program of the Chinese Academy of Sciences (JH2022007).

## CONFLICT OF INTEREST STATEMENT

We declare no conflicts of interest.

## Supporting information


Data S1.

Table S1.

## Data Availability

Results of MR studies are available from each published study.
