# Supplementary material for: Association between genetically determined telomere length and health‐related outcomes: A systematic review and meta‐analysis of Mendelian randomization studies
Source: Aging Cell. 2023 May 26;22(7):e13874. doi: 10.1111/acel.13874 (PMC10352568; doi:10.1111/acel.13874)
Supplement: Supplementary file 1 — Data S1. [file ACEL-22-e13874-s001.docx]

**Supplementary Methods**

**Search terms to identify relevant studies in PubMed, EMBASE, and Web of Science**

PubMed

#1 Leukocyte telomere length

("telomere"[MeSH Terms]) OR ("telomere"[All Fields]) OR ("telomere length" [All Fields]) OR ("leukocyte telomere length" [All Fields]) OR ("LTL" [All Fields])

#2 Mendelian randomization

("Mendelian randomization analysis"[MeSH Terms]) OR ("Mendelian randomization" [All Fields]) OR ("MR"[All fields]) OR ("instrumental variable analysis"[All Fields])

1 AND 2: 386 results (13-04-2022)

EMBASE

#1 Leukocyte telomere length

("telomere*" or "telomere length" or "leukocyte telomere length" or "LTL").af.

#2 Mendelian randomization

("mendelian randomization analysis" or "mendel* random*" or "MR" or "instrumental variable analysis").af.

1 AND 2: 619 results (13-04-2022)

Web of Science

#1 drugs

TS=(("telomere") OR ("telomere length") OR ("leukocyte telomere length") OR ("LTL"))

#2 Mendelian randomization

TS=(("mendel* random*") OR ("MR") OR ("instrumental variable analysis"))

1 AND 2: 240 results (13-04-2022)
